# Ultra-sensitive molecular detection of gene fusions from RNA using ASPYRE

**DOI:** 10.1186/s12920-022-01363-0

**Published:** 2022-10-12

**Authors:** Eleanor R Gray, Justyna M Mordaka, Efthimia R Christoforou, Kristine von Bargen, Nicola D Potts, Christina Xyrafaki, Ana-Luisa Silva, Magdalena Stolarek-Januszkiewicz, Katarzyna Anton, Paulina K Powalowska, Simonetta Andreazza, Alessandro Tomassini, Rebecca N Palmer, Aishling Cooke, Robert J Osborne, Barnaby W Balmforth

**Affiliations:** Biofidelity Ltd, 330 Cambridge Science Park, Milton road, CB4 0WN Cambridge, England

**Keywords:** RNA, Gene fusions, NSCLC, Sensitive, Molecular detection

## Abstract

**Background:**

RNA is a critical analyte for unambiguous detection of actionable mutations used to guide treatment decisions in oncology. Currently available methods for gene fusion detection include molecular or antibody-based assays, which suffer from either being limited to single-gene targeting, lack of sensitivity, or long turnaround time. The sensitivity and predictive value of next generation sequencing DNA-based assays to detect fusions by sequencing intronic regions is variable, due to the extensive size of introns. The required depth of sequencing and input nucleic acid required can be prohibitive; in addition it is not certain that predicted gene fusions are actually expressed.

**Results:**

Herein we describe a method based on pyrophosphorolysis to include detection of gene fusions from RNA, with identical assay steps and conditions to detect somatic mutations in DNA [1], permitting concurrent assessment of DNA and RNA in a single instrument run.

**Conclusion:**

The limit of detection was under 6 molecules/ 6 µL target volume. The workflow and instrumentation required are akin to PCR assays, and the entire assay from extracted nucleic acid to sample analysis can be completed within a single day.

**Supplementary Information:**

The online version contains supplementary material available at 10.1186/s12920-022-01363-0.

## Background

As precision oncology becomes routine, identification of patients most likely to benefit from targeted therapeutics requires a diagnostic technology with fast turnaround time, low-cost and appropriately sized panels of relevant targets necessary for timely clinical decision-making, for both first-line treatments and at progression. RNA is an under-utilised but critical analyte for oncology, and contains information on transcript variations and expression levels, and is affected by genomic changes outside of exons [2, 3]. Gene fusions commonly occur in intronic regions, leading to aberrant splicing of two independent genes, and are enriched in certain sub-populations of patients with non-small cell lung carcinoma (NSCLC; e.g. young/never-smokers) [4]. Targeted therapies are indicated for several common gene fusions in NSCLC including *ALK*, *ROS1*, and *RET*, making this class of mutations critical for clinical decision making, but robustly and accurately identifying these patients remains challenging using current technology. Most commercially available targeted next generation sequencing (NGS) methods that analyse variations in exons from genomic material enable many common DNA mutations to be detected, but fail to provide a full dataset for actionable RNA mutations, as they rely on DNA-based sequencing to infer intronic mutations that are predicted to lead to fusion events manifest in the RNA [5, 6]. The intronic regions are large and full of repetitive sequences, and hence require sequencing depths that are costly and require large amounts of tissue (for sequential sequencing) in addition to comprehensive predictive algorithms, and/or RNA-based subsequent sequencing, leading to poor diagnostic accuracy of predicted fusion breakpoints. Taken together, exhaustion of samples due to sequential analysis of multiple DNA and RNA biomarkers makes it imperative that a workflow using DNA and RNA co-extraction from samples is employed for efficient biomarker detection to allow for fully-informed clinical decision-making.

Previously, we demonstrated that detection of a panel of somatic variants in DNA from tumor tissue was possible down to single-molecule levels of sensitivity by using a simple, fast and low-cost method incorporating Allele-Specific PYrophosphorolysis REaction (ASPYRE) [1]. Herein we show further capabilities of the ASPYRE technology by detecting gene fusions from RNA, showing single-molecule detection limits equivalent to DNA, scaling to over 30 fusion targets from one initial RT-PCR amplification reaction. The same steps, equipment and programs are used for both RNA and DNA, permitting parallel assessment in a single instrument run. The ASPYRE-Lung RNA panel consists of 37 common actionable fusion targets relevant to NSCLC including *ALK*, *ROS1*, *RET*, MET exon 14 skipping, and *NTRK1*, *NTRK2* and *NTRK3* (Supplementary Table 1). We show detection of targets in commercially sourced synthetic samples, with detection down to single molecule copy numbers. High specificity is shown using normal formalin-fixed paraffin-embedded (FFPE) lung tissue samples with a final demonstration of end-to-end execution of our assay from lung tissue to result using confirmed RNA fusion-positive FFPE samples.

## Methods

### Reference samples

The 18-plex Seraseq Fusion RNA Mix v4 reference standard sample was purchased from LGC Seracare (Milford, USA). Synthetic RNA oligonucleotides were manufactured by GenScript (Leiden, NL), and diluted in 0.2 µg/µL polyA carrier RNA (Qiagen, Manchester, UK). Purified total lung RNA (AM7968) was obtained from ThermoFisher Scientific (Waltham, USA) and used as background RNA.

### Clinical samples

De-identified samples supplied by biobanks were from sites worldwide, collected under local IRB approved informed consents or through waiver of consent after 10 years storage, consistent with IRB and Ethics Committee guidelines. Lung tissue FFPE blocks from normal lung tissue were procured from Discovery Life Sciences, Santa Barbara, USA and AMS Bio, Abingdon, UK. Blocks were sectioned using a microtome, producing three curls of 12 µM per replicate. Lung tissue FFPE curls from patients with confirmed RNA fusions were obtained from Azenta Life Science (Laval, Canada).

### Nucleic acid extraction

RNA and DNA was extracted using the parallel protocol with the Quick-DNA/RNA™ FFPE miniprep kit (Zymo Research, Irvine, USA) and stored at -20 °C until analysis. Nucleic acid concentrations were measured using Qubit™RNA and DNA High Sensitivity kits (ThermoFisher).

### ASPYRE

Target amplification, enzymatic digestion, pyrophosphorolysis and ligation, and rolling circle amplification were carried out essentially as described in Silva et al. with modifications described below:

The PCR mastermix from [1] was modified to perform RT-PCR in 6 µL of mixture per reaction by the addition of 1x Luna WarmStart reverse transcriptase (E3006, NEB) and the reaction performed in 1x Q5U buffer (M0515, NEB) with 2 mM MgSO_4_ (NEB), and 6 µL of RNA target. The thermocycling conditions used were 37 ˚C 1 min; 55 ˚C 10 min; 98 ˚C 1 min; 50 cycles of 98 ˚C 10 s, 63 ˚C 15 s, 72 ˚C 15 s; and 72 ˚C 5 min. Proteinase K digestion and pyrophosphorolysis were performed essentially as described previously [1]. For the RCA, 10 µL of detection mixture per reaction was prepared by mixing 1x RCA buffer (51.8 mM Tris-HCl pH 8.8, 27.6 mM NH_4_SO_4_, 3.72 mM KCl, 3.49 mM MgSO_4_, 0.0567% Tween-20), 300 U/mL *Bst* 3.0 WarmStart (M0374, NEB), 0.8 mM dNTPs with dUTP (Promega), 0.3% polydimethylsiloxane emulsifier (A5757, Sigma Aldrich), and 0.253 µM primer mix. 5 µL PPL reaction sample was added to 10 µL of detection mix in a 384-well plate. Samples were incubated at 57 °C for 200 min in a QuantStudio5 RealTime PCR System (ThermoFisher). The fluorescence read-out was taken every minute in the FAM, JUN, VIC and Cy5 channels.

### Digital RT-PCR

Quantification of synthetic RNA oligonucleotides by RT-dPCR was performed in a QIAcuity One (Qiagen) using the RT-PCR mix from the ASPYRE assay with the following additions: 0.1% Tween-20 (Sigma-Aldrich), EVAgreen (Biotium) 3X, AlexaFluor 700 (ThermoFisher) 0.3 ng/µl. The number of amplification cycles was reduced to 40.

### Data Analysis

A baseline correction was applied to fluorescence data to correct for drift, and Cycle Sigmoid midpoint (CSm) values were identified using fits similar to those described previously [7]. Thresholds were set for each of the variants at the lower value of either five standard deviations below the mean of the NTC reaction or ten standard deviations above the mean of the positive control samples. Poisson sampling was used to determine the theoretical probability of a mutation being present at each number of copies. To assess the relationship between theoretical and experimental limiting dilution series, we performed non-parametric Spearman’s rank correlation tests. We adopted a significance criterion □ = 0.05 and type I error was controlled using Benjamini and Hochberg false discovery rate (FDR) correction for multiple comparisons.

For statistical analyses we used the statistical software package R (http://www.r-project.org/) and the ‘tidyverse’ library for data organisation and visualisation [8].

## Results

### Detection of reference standard RNA targets by the ASPYRE assay

The ASPYRE assay comprises four simple sequential enzymatic steps, requiring only reagent transfer. The stages were described previously for DNA: 1) PCR, 2) enzymatic cleanup, 3) exonuclease digestion, hybridisation, pyrophosphorolysis (PPL), and ligation; and 4) rolling circle isothermal amplification (RCA; Supplementary Figs. 2 and [1]). To adapt this method to incorporate RNA as analyte, we added a reverse transcriptase (RT) step to the initial PCR, and created a pool of primers to amplify 37 gene fusions in which the 3’ partners are *ALK* exon 20, *ROS1* exons 32, 34, 35, and 36, *RET* exons 8, 11 and 12, *MET* exon 14 skipping, *NTRK1* exon 10, *NTRK2* exon 14, and *NTRK3* exons 14 or 15 (Supplementary Table 1). The forward primer targets the 5’ partner of these genes, engendering the specificity of the reaction since only samples with a gene fusion present will exponentially amplify.

For analysis of mutations from DNA, probes are designed to cover the site of mutation. Conversely, for analysis of gene rearrangements from RNA, probes can be designed to either match the exon boundary formed when two exons fuse together, or to common sequences of a family of gene fusions. During the third stage of the ASPYRE assay, only probes perfectly matched to target fusion sequences are fully digested by pyrophosphorolysis to the point at which they can be ligated to form a circular ssDNA, as described previously (Supplementary Figs. 2 and [1]). The resulting circularised probes act as a template for subsequent isothermal amplification. Current standard of care guidelines for NSCLC from NCCN, ESMO, and CAP/IASLC/AMP recommend testing for rearrangements of 6 genes, and exon skipping events in 1 gene [9]. Detection of fusion events across all these genes using ASPYRE can be achieved using only 2 wells, enabling testing of large numbers of samples in parallel in a single real-time PCR instrument run. Detection of mutations from DNA across practice guideline recommended markers in *EGFR*, *KRAS*, *BRAF*, and *ERBB2* can be analyzed in 20 wells ([1] and data not shown). We adapted our previous isothermal amplification reaction to multiplex four colors in each well, enabling detection of all RNA targets into two reaction wells with each colour indicating a 3’ gene family class of target. Guidelines for treatment of gene fusions are 3’-specific, with no discrimination between 5’ partners, thus the outcome is read as *ROS1*-positive, *ALK*-positive, or similar [9].

**Fig. 1:**
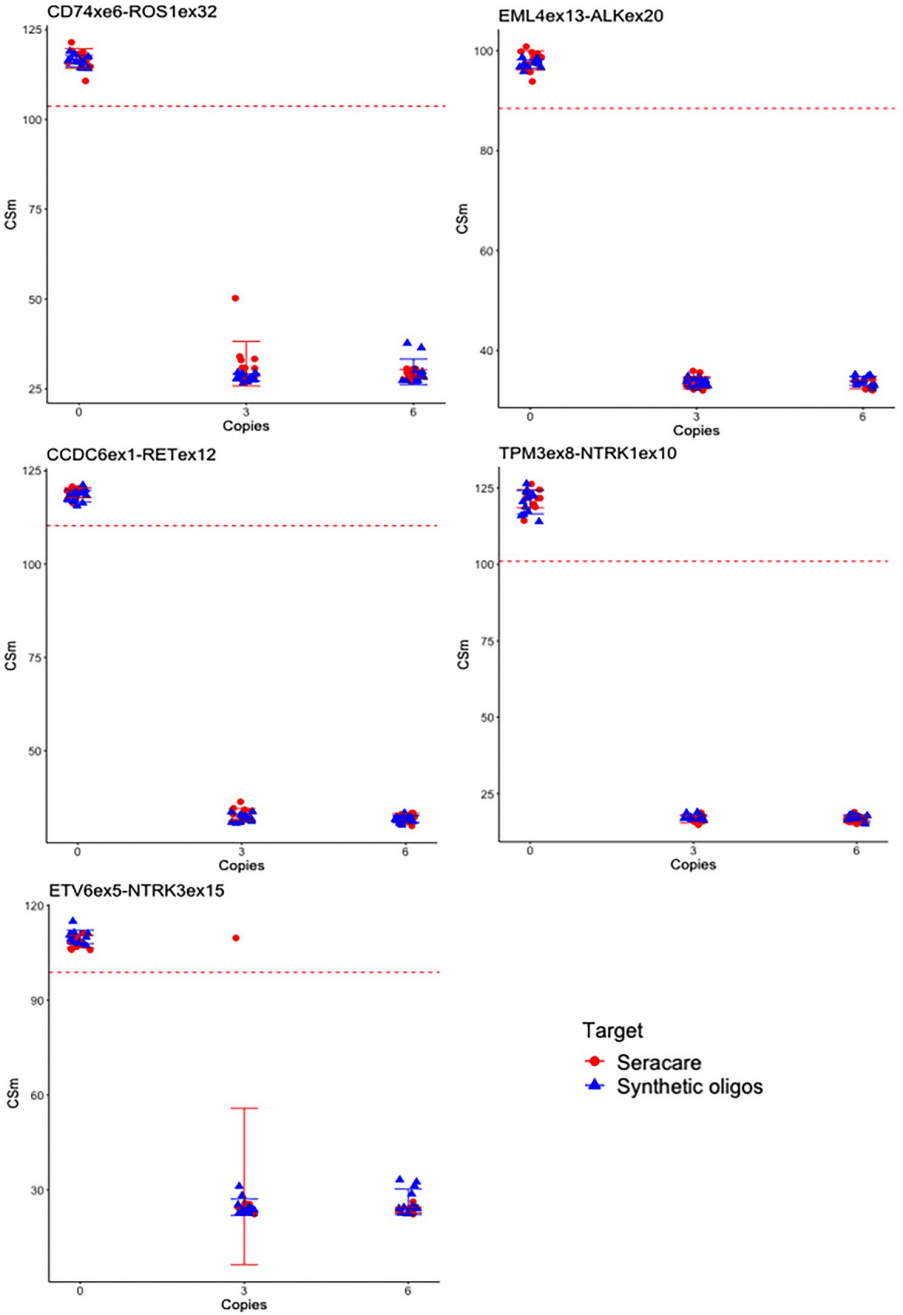
Detection of five RNA targets by the ASPYRE assay *(CD74ex6-ROS1ex34, EML4ex13-ALKex20, CCDC6ex1-RETex12, TPM3ex8-NTRK1ex10* and *ETV6ex5-NTRK3ex15).* Samples were derived from Seracare RNA Fusion v4 mix, or made in-house from synthetic oligonucleotides and diluted into total lung RNA as a background. Each data point represents a single replicate (12 per input level at zero, three or six copies of RNA oligonucleotide); means and standard deviations are also shown

Since several common fusion targets were not available as commercial reference standards, we sought to benchmark our in-house panel of synthetic RNA oligonucleotides to those from a commercial reference standard to validate assay performance. RNA oligonucleotides were diluted into poly-A carrier RNA (cRNA) and quantified by digital RT-PCR. The commercial reference standard Seraseq Fusion RNA mix v4 contains a mix of 18 RNA targets quantified by dPCR by the supplier. To confirm the quality and validity of our dRT-PCR quantified samples, a comparison of the outcomes of the ASPYRE-Lung RNA assay with single primers and probes against a selection of five targets using both the in-house quantified RNA oligos and the Seraseq Fusion mix was performed. The results (Fig. 1) are shown as Cycle Sigmoid midpoint (CSm), whereby the result at zero copies represents the background signal arising from non-specific amplification of the probe [1, 10]. The curves that yield these CSm data are shown in Supplementary Fig. 1. The ASPYRE technology yields a qualitative result, whereby a CSm of less than the threshold indicates a positive result for the presence of each variant of interest. Here, the threshold is set at five standard deviations below the mean of the negative (background) control. There was no difference in mean CSm between the two target source types (Seracare or dRT-PCR-quantified synthetic oligonucleotides). All CSm values for samples that contained target molecules were below the threshold apart from a single replicate for ETV6-NTRK3 (Fig. 1). With an average of three copies per well, stochasticity results in a 5% chance that a replicate well contains zero copies, consistent with these results. These results demonstrate equivalency of our synthetic reference material compared to reference standards.

### Testing the ASPYRE assay performance for detection of RNA fusions

**Fig. 2:**
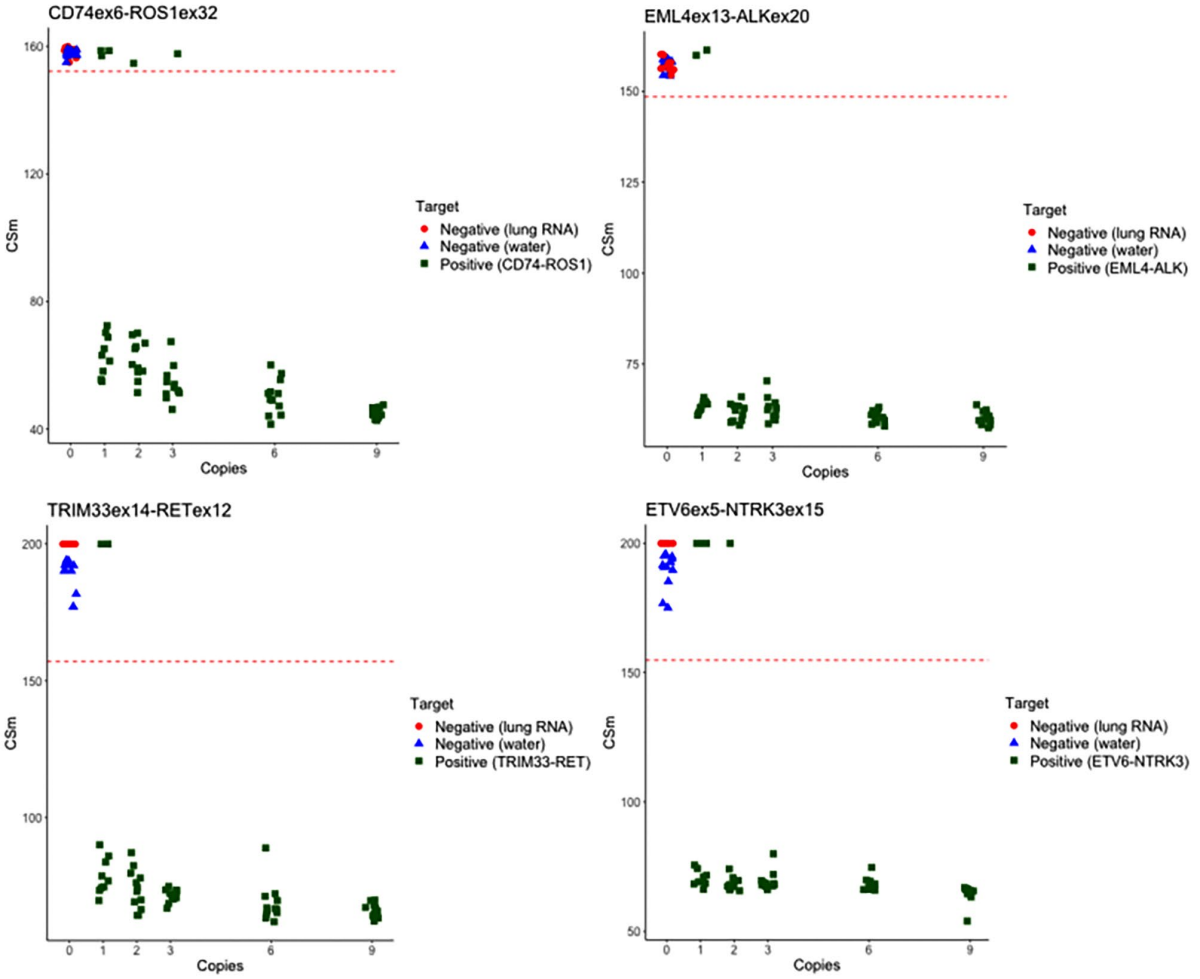
The ASPYRE-Lung RNA assay approaches the single molecule limit. Synthetic RNA oligonucleotides were quantified by dPCR, and added to reactions at the specified number of copies, with 12 replicates. Detection of one, two, three, six or nine copies of target in the multiplexed ASPYRE-Lung assay, for the following targets: *EML4ex13-ALKex20, ETV6ex5-NTRK3, CD74ex6-ROS1ex32*, and *TRIM33ex14-RETex12.* Two negative controls were also analyzed: water, and total human lung RNA.

Following demonstration of equivalence to commercial RNA reference standards, we used our synthetic RNA oligonucleotides to test a wider range of targets relevant to NSCLC. The full ASPYRE-Lung RNA panel uses multiplexed primers in the initial RT-PCR reaction, and multiplexed probes in the PPL and RCA steps to detect any of 37 potential targets (Supplementary Table 1) within the 3’ gene fusion families. Altogether these families collectively account for the approximately 11.3% of NSCLC cases that are gene-fusion positive [11]. We tested the analytical sensitivity of our multiplexed reactions on synthetic oligonucleotide targets representing four classes of gene fusions detected by ASPYRE: *ROS1*, *ALK*, *RET* and *NTRK*. Unlike DNA targets, for which analysis of different ratios of wild-type to mutated DNA can be used to define a limit of detection, RNA gene fusions have no equivalent wild-type background, and are therefore detected as a binary present/absent result. We assessed the assay limit of detection as the number of amplifiable copies of fusion target, using limiting dilution. Synthetic RNA oligonucleotide targets were added to RT-PCR reactions at zero, one, two, three, six or nine average copies per reaction, with twelve replicates. At a copy number of three, six or nine target molecules per reaction, all repeat wells were positive for all targets, which is indicative of the ASPYRE assay being able to detect as low as three copies per six microlitres. Poisson sampling at 2 copies or fewer per reaction results in greater than a 1/12 chance that a reaction will contain 0 copies and give a negative result (Fig. 2 and Supplementary Table  2). Indeed, stochastic sampling results in some wells without any target which is reflected in the negative results seen at one or two copies of target molecules per reaction.

Supplementary Table  2 compares the results to a theoretical distribution assuming a reaction efficiency of 100% at the copy numbers given. The correlation of experimental and theoretical results were significant at P = 0.029 (*ALK and RET: rho = 0.857* ), P = 0.01 (*ROS1: rho = 0.985*) and P = 0.06 (*NTRK: rho = 0.955*), demonstrating that detection using ASPYRE is consistent with single molecule detection.

### Testing the specificity of the ASPYRE-Lung RNA panel

We next tested the specificity of the ASPYRE-Lung RNA panel using FFPE samples from normal lung tissue. Five FFPE blocks containing normal lung tissue were sourced from two biobanks, and four 10–15 µM curls sectioned from each block. Block source data are shown in Supplementary Table 3. Nucleic acid was purified and quantified from each curl independently; and 1, 5 or 10 ng RNA processed through the ASPYRE assay by two independent users. The range of RNA inputs used was chosen both to showcase the sensitivity of the assay, at 1 ng, and to match the lowest input requirement of comparable technologies, at 10 ng. The results from ASPYRE for each 3’ fusion partner class are shown in Supplementary Table 4. ACTβ (β-actin) was used as a positive control to ensure that detectable levels of RNA were added to the reaction, and gave a positive result for all replicate curls of all tissue samples for both users, except one at 10 ng probably due to pipetting error. Under standard operating procedures this would trigger an invalid result, and reanalysis. The overall analysis returned negative results for all replicate curls of all samples for both users across all RNA fusions, as expected for samples that contained normal lung tissue, with a negative predictive value of 100%.

### Detection of gene fusions from patient tissue-derived RNA

**Fig. 3:**
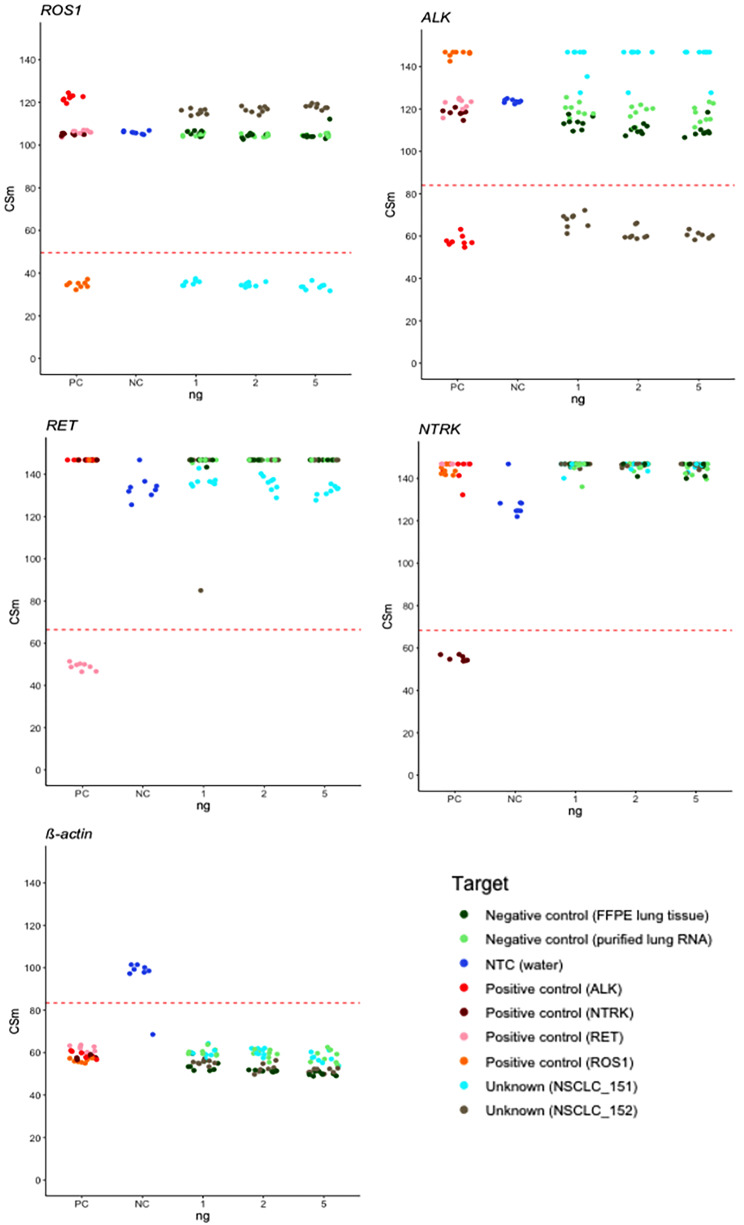
Analysis of two known gene fusion-positive FFPE samples by the ASPYRE-Lung assay. Shown are the CSm signals obtained for the different RNA fusion classes tested in the assay: *ROS1, ALK, RET, NTRK*, and positive control (*ACTβ*). Red lines indicate thresholds that distinguish positive from negative calls; a CSm value under the threshold is positive

Finally, we demonstrate concordance with orthogonal detection methods using FFPE lung tumor tissue samples from patients with known gene fusions. The entire ASPYRE-Lung workflow for RNA from sample to result was applied to two FFPE lung tumor samples with confirmed RNA fusions (one *ROS1*-positive, and one *ALK*-positive) previously detected via fluorescence *in-situ* hybridization (FISH) and immunohistochemistry (IHC) respectively. RNA was extracted from curls, and sequential input amounts (1, 2 and 5 ng per reaction) were processed through our standard multiplexed ASPYRE-Lung fusion assay (Fig. 3). Alongside the two patient tumor samples, positive controls (synthetic oligonucleotides) from each of four gene fusion classes were also tested at 6 copies each (tested in a background of total lung RNA), as well as negative controls (FFPE normal lung tissue and total lung RNA), and no-template controls (water).

Sample NSCLC_151 (expected *ROS1*-positive) gave a positive signal commensurate with the *ROS1*-positive control at all input levels of RNA tested (Fig. 3), and consistent with the *ACTβ* RNA positive control. It was negative for *ALK, RET* and *NTRK* classes of gene fusions. Sample NSCLC_152 (expected *ALK*-positive) gave a positive signal commensurate with the *ALK*-positive control at all input levels of RNA tested (Fig. 3). It was negative for other classes of fusions tested, including *ROS1*, *RET* and *NTRK*. A single no-template control (water) out of the eight performed yielded a false-positive result for *ACTβ*. Under standard operating procedures this would trigger an invalid result for the plate, and reanalysis.

Taken together, these data demonstrate concordance between the ASPYRE-Lung assay and previous orthogonal testing for confirmed RNA fusion-positive samples using the end-to-end ASPYRE workflow. Extraction of nucleic acid from the two samples and execution of the four stages of the assay from curl to result was easily completed in 1.5 workdays, accommodating multiple stopping points to facilitate combining the workflow with laboratory shift patterns and other workstreams.

## Discussion

Across 102 countries, under half of patients diagnosed with lung cancer have access to molecular testing analysis at all [12]. For those who are able to access NGS prior to first line treatment, the average turnaround time to result can stretch to weeks [13–15]. As such, almost a fifth of patients begin standard of care cytotoxic chemotherapy, before receiving results from biomarker testing [16]. Moreover, full molecular analysis of all targets is frequently limited to those who access and enroll in clinical trials, with resulting high disparities in outcomes by socioeconomic and race brackets [17]. Standard assays for detecting gene fusions in current clinical usage include those based on IHC, FISH, and NGS (Table 1) [18, 19]. While IHC and FISH are established methods, the inability to test for all classes of potential gene fusions across a wide range of input calls limits their use. In addition, these tests are not high throughput and there is inter-operator variability on the interpretation of staining. In particular, diagnosis of *ROS1* or *NTRK* fusions by IHC should be confirmed with FISH due to weak background or baseline staining and false-positives in patients who are (ex-)smokers, incurring delays with each test required [9]. NGS targeted methods to detect gene fusions that use DNA are based on detection of translocations in intronic regions and prediction of whether these will lead to aberrant transcripts, which can be highly problematic given the heterogeneity of these kinds of events, though useful as a secondary discovery tool alongside assays with a more rapid turnaround time for clinical decision making. Interpretation of NGS data requires extensive bioinformatics support, which is not readily accessible in community oncology settings in the timeframes required, and can leave health care professionals unsure of how to interpret results [12, 20]. RNA-based approaches can be limited by the quantity and quality of RNA required, with techniques based on RT-PCR limited to at most a handful of gene targets which fail to cover the diversity of potential 5’ fusion partners required [21].

In our previous study introducing the ASPYRE technology, we reported the design of multiplex panels to target somatic variations in DNA that can detect mutations at single molecule levels. Herein we extend the capabilities of ASPYRE to include detection of targets from RNA, specifically gene fusions in groups including 3’ partners of *ALK*, *ROS1*, *RET* and *NTRK1*, *NTRK2* and *NTRK3*. No changes were made to the ASPYRE workflow, with the only adaptation being the addition of a reverse transcriptase to one mastermix formulation. As a result, DNA and RNA samples can be run concurrently with no additional steps required from the user, on the same plate in the same thermal cycler and qPCR machine. The entire post-extraction workflow, from RT-PCR through to result can be completed in under a day. Up to 192 samples can be processed through this fusion assay in a single real-time PCR instrument run, or 16 samples when combined with the 20 wells used for detection of mutations from DNA (data not shown).

A combination of multiplexed primers (25 forward, and 12 reverse) allows amplification of all targets in our RNA panel to be run from a single RT-PCR reaction. This panel includes all 3’ partner classes of actionable mutations listed in current NSCLC treatment guidelines (NCCN, ASCO/CAP, ESMO) for advanced/metastatic disease. Collectively, these targets represent just over 11% of reported 3’ classes of mutations found as RNA gene fusions in NSCLC [11]. The probes used in the hybridisation and pyrophosphorolysis step for gene fusions with multiple 5’ partners to common 3’ partner exons are complementary to the 3’ fusion partner exon alone, enabling multiple 5’ partners to be detected with a single, common probe. This assay design also facilitates addition of new 5’ partners as they are reported, with minimal adjustment required to expand the included targets. Our assay has been optimised to detect down to six input copies per six microliters of the RNA targets, and has a limit of detection approaching or achieving single molecule detection limits, making it a powerful tool for clinical situations where only limited material is available. This sensitivity does not come at a cost to specificity, as no false-positives were reported across repeat analyses of normal lung tissue controls. Finally, two lung tumor FFPE samples from patients with confirmed gene fusion-positive NSCLC were used to demonstrate concordance with orthogonal testing methods and end-to-end assay performance, from sample through extraction and the four steps of ASPYRE to the end result. The two samples gave consistently positive results for the expected gene fusion class, even when as little as 1 ng input was used. This demonstrates the potential for use with samples of low cellularity and correspondingly low yields of RNA, with no assay inhibition from carryover of contamination from the FFPE samples at any input level.

## Conclusion

RNA is an under-utilised resource for diagnostic testing of patients with NSCLC; yet the availability of targeted therapy for specific gene fusions renders the need urgent for accurate, fast, cost-effective diagnostics that are scalable and easy to integrate into existing laboratory workflows. Access to a simple, fast, local and low-cost panel that can provide a genotype for all actionable mutations of NSCLC would be transformative for patients. Equally, a panel that does not require extensive investment in specialised equipment and training for end-users and care providers to implement into routine clinical workflow to improve patient outcomes would be practice changing. We expect that the ASPYRE technology has the potential to transform current care pathways, both for NSCLC and future oncology applications.

**Table 1:** A comparison of frequently used analysis methods to detect gene fusions with the ASPYRE assay ([22–24] and manufacturer’s guidelines)

	NGS	ASPYRE	RT-PCR	IHC	FISH
Method performance characteristics	Highly variable performance due to need to sequence large intronic regions	< 6 copies/6 µL (Analytical sensitivity)	Extremely high diagnostic sensitivity	Variable performance; subject to interobserver variability	Variable performance; subject to interobserver variability
Multiplexing	1000+	37	< 10	1 3’ gene	1 3’ gene
Prior knowledge of 5’ & 3’ fused genes required	No	Yes	Yes	No	No
Instrument complexity	High	Low	Low	Moderate	Moderate
Laboratory workflow complexity	High	Medium	Medium	Medium	High
Data analysis complexity	High	Low	Low	Medium	Medium
Time to result	> 7 days	1–2 days	1–2 days	2–3 days	2–3 days
Material required	10–55 ng	1–10 ng	> 10 ng	2 slides per stain	2 slides
Cost	High	Low	Medium	Low	Medium

## Electronic supplementary material

Below is the link to the electronic supplementary material.

Supplementary Material 1

## Data Availability

The datasets used and/or analysed during the current study are available from the corresponding author on reasonable request.

## Declarations

Discovery Life Sciences (USA): Two (HBF_0018 and HBF_0019) are from autopsies performed in 2017, collected under informed consent. HBF_022 was collected under waiver of consent: after 10 years storage under IRB approval to use such specimens for research by waiver of consent per federal regulations as long as the FFPE block has met the 10-year storage requirement.

Azenta Life Sciences (France): NSCLC_151 and NSCLC_152. Article L.1131-1-1 of the public health code of French law explicitly allows opt-out consent where genetic analysis is based on the reuse of already collected samples. The patient was informed through an information notice of the use of their samples for research and was able to object to this. The protocols and regulations of Cypath (supplier to Azenta Life Sciences) have been approved by the French Ministry of Health and Research (declaration number: DC-2020-4290, cession authorization number AV-2020-4288).
