# Supplementary material for: Ultra-sensitive molecular detection of gene fusions from RNA using ASPYRE
Source: BMC Med Genomics. 2022 Oct 12;15:215. doi: 10.1186/s12920-022-01363-0 (PMC9555097; doi:10.1186/s12920-022-01363-0)
Supplement: Supplementary file 1 — Supplementary Material 1 [file 12920_2022_1363_MOESM1_ESM.docx]

**Title: Ultra-sensitive molecular detection of gene fusions from RNA using ASPYRE**

Author list: Eleanor R Gray, Justyna M Mordaka, Efthimia R Christoforou, Kristine von Bargen, Nicola D Potts, Christina Xyrafaki, Ana-Luisa Silva, Magdalena Stolarek-Januszkiewicz, Katarzyna Anton, Paulina K Powalowska, Simonetta Andreazza, Alessandro Tomassini, Rebecca N Palmer, Aishling Cooke, Robert J Osborne, Barnaby W Balmforth*

Affiliations: Biofidelity Ltd, 330 Cambridge Science Park, Milton road, Cambridge, CB4 0WN

**Supplementary Information**

| **5’ Gene fusion** | | **3’ Gene fusion** | | **Alternative name** | **COSMIC ID** |
| --- | --- | --- | --- | --- | --- |
| **Gene** | **Exon** | **Gene** | **Exon** |  |  |
| ***EML4*** | 13 | ***ALK*** | 20 | Variant 1 | COSF408 |
|  | 20 |  |  | Variant 2 | COSF409 |
|  | 6 |  |  | Variant 3a | COSF411 |
|  | 6ins33 |  |  | Variant 3b | COSF474 |
| ***KIF5B*** | 24 |  |  |  | COSF1058 |
| ***EML4*** | 6 |  | 20ins18 |  | COSF1544 |
|  | 20 |  |  |  | COSF730 |
| ***KIF5B*** | 15 | ***RET*** | 12 |  | COSF1232 |
|  | 16 |  |  |  | COSF1230 |
|  | 22 |  |  |  | COSF1253 |
|  | 23 |  |  |  | COSF1234 |
| ***CCDC6*** | 1 |  |  |  | COSF1271 |
| ***NCOA4*** | 6 |  |  |  | COSF1341 |
| ***TRIM33*** | 14 |  |  |  | ND |
| ***KIF5B*** | 24 |  | 11 |  | COSF1262 |
|  |  |  | 8 |  | COSF1242 |
| ***TPM3*** | 8 | ***NTRK1*** | 10 |  | COSF1329 |
| ***QKI*** | 6 | ***NTRK2*** | 14 |  | COSF1446 |
| ***ETV6*** | 5 | ***NTRK3*** | 15 |  | COSF571 |
|  | 4 |  | 14 |  | COSF1534 |
| ***MET*** | 13 | ***MET*** | 15 |  | COSM13245 |
| ***CD74*** | 6 | ***ROS1*** | 32 |  | COSF1202 |
| ***SLC34A2*** | 4 |  |  | Variant 1 | COSF1196 |
|  | 13del |  |  | Variant 3 | COSF1259 |
| ***SDC4*** | 2 |  |  |  | COSF1265 |
|  | 4 |  |  |  | COSF1278 |
| ***CD74*** | 6 |  | 34 |  | COSF1200 |
| ***SLC34A2*** | 4 |  |  | Variant 2 | COSF1198 |
|  | 13del |  |  | Variant 4 | COSF1261 |
| ***EZR*** | 10 |  |  | Variant 1 | COSF1267 |
| ***SDC4*** | 2 |  |  |  | COSF1671 |
|  | 4 |  |  |  | COSF1280 |
| ***GOPC*** | 8 |  | 35 | Variant 3 | COSF1139 |
| ***LRIG3*** | 16 |  |  |  | COSF1269 |
| ***TPM3*** | 8 |  |  | Variant 1 | COSF1273 |
| ***CD74*** | 6 |  |  |  | COSF1478 |
| ***GOPC*** | 4 |  | 36 | Variant 2 | COSF1188 |

**Supplementary Table 1:** Gene fusions detectable through the RNA panel of the ASPYRE-Lung assay. Indicated are the 5’ and 3’ gene names and exons, and the corresponding identifier in the COSMIC database. ND – not described.

| **Theoretical copy no. per reaction** | **Expected no. positives /12** | ***ROS1*** | ***ALK*** | ***RET*** | ***NTRK*** |
| --- | --- | --- | --- | --- | --- |
| 0 | 0 | 0 | 0 | 0 | 0 |
| 1 | 7.6 ± 1.7 | 9 | 10 | 9 | 8 |
| 2 | 10.4 ± 1.2 | 11 | 12 | 12 | 11 |
| 3 | 11.4 ± 0.8 | 11 | 12 | 12 | 12 |
| 6 | 12.0 ± 0.2 | 12 | 12 | 12 | 12 |
| 9 | 12 ± 0.03 | 12 | 12 | 12 | 12 |

**Supplementary Table 2**. Limiting dilution series tested by the ASPYRE-Lung RNA panel. The number of positive reactions out of 12 at each concentration is shown, derived from the data shown in Figure 2.

| **Sample ID** | **Source Biobank** | **Pathology diagnosis** | **Tissue** | **Age** | **Gender** | **Ethnicity** | **Necrosis (%)** | **Tumor content (%)** |
| --- | --- | --- | --- | --- | --- | --- | --- | --- |
| HBF_0013 | AMS Bio | Normal | Lung | 71 | M | Caucasian | <20 | n/a |
| HBF_0014 | AMS Bio | Normal | Lung | 60 | M | Caucasian | <20 | n/a |
| HBF_0018 | Discovery Life Sciences | Normal | Lung | 65 | F | White | 0 | n/a |
| HBF_0019 | Discovery Life Sciences | Normal | Lung | 74 | M | White | 0 | n/a |
| HBF_0022 | Discovery Life Sciences | Normal | Lung | NK | NK | NK | 0 | n/a |
| NSCLC_151 | Azenta Life Science | NSCLC - Adenocarcinoma | Lung | 42 | F | NK | 0 | 30 |
| NSCLC_152 | Azenta Life Sciences | NSCLC - Adenocarcinoma | Lung | 80 | M | NK | 0 | 30 |

**Supplementary Table 3:** Patient data for clinical samples used in this study. All clinical data were supplied by the corresponding biobank. NK – not known. Sample NSCLC_151 was identified as *ROS1*-translocation positive by FISH, and NSCLC_152 as *ALK*-translocation positive by IHC (D5F3).

| Sample input | | User 1 | | | | | User 2 | | | | |
| --- | --- | --- | --- | --- | --- | --- | --- | --- | --- | --- | --- |
| Sample | Replicates | *ALK* | *ROS1* | *RET* | *NTRK* | *ACTβ* | *ALK* | *ROS1* | *RET* | *NTRK* | *ACTβ* |
| Water | 8 repeats | - | - | - | - | - | - | - | - | - | - |
| HBF_0013 | 1-4 | - | - | - | - | + | - | - | - | - | + |
| HBF_0014 | 1-4 | - | - | - | - | + | - | - | - | - | 3+  1- |
| HBF_0018 | 1-4 | - | - | - | - | + | - | - | - | - | + |
| HBF_0019 | 1-4 | - | - | - | - | + | - | - | - | - | + |
| HBF_0022 | 1-4 | - | - | - | - | + | - | - | - | - | + |

**Supplementary Table 4:** Analysis by the ASPYRE-Lung RNA assay of 20 different input samples by two independent users. Samples comprised four replicate curls taken sequentially from five FFPE lung tissue resections from patients with normal lung tissue. Results represent all repeats: ‘-’ negative, ‘+’ positive; unless otherwise indicated. One false negative repeat was seen for one sample (NBF_0014) for *ß-actin*, possibly from pipetting error, and would under standard testing conditions trigger re-analysis of the sample.


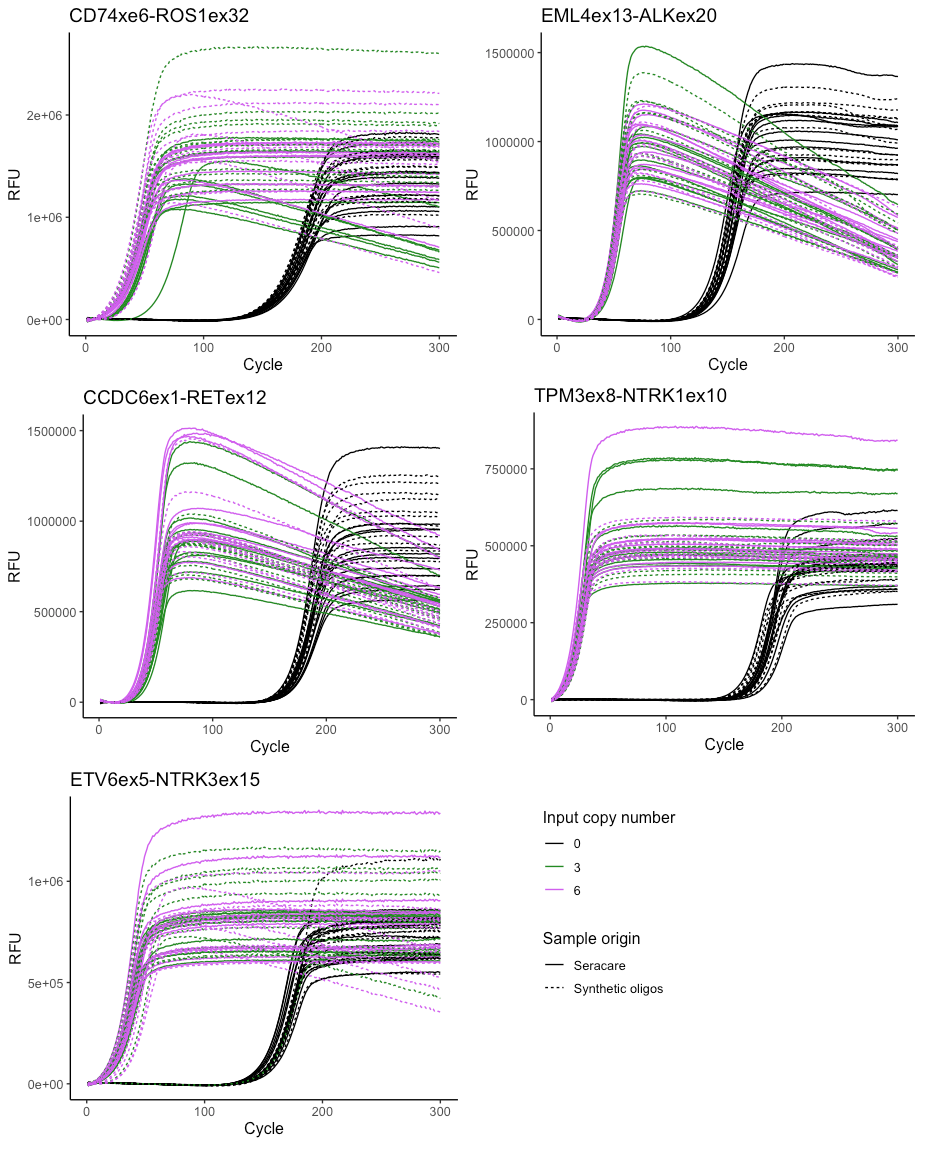


**Supplementary Figure 1:** Amplification data from which CSm values shown in Figure 1 are derived. Shown are the curves for zero, three or six copies of target for *CD74ex6-ROS1ex34, EML4ex13-ALKex20, CCDC6ex1-RETex12, TPM3ex8-NTRK1ex10* or *ETV6ex5-NTRK3ex15*. The target source is indicated by a dashed line (synthetic oligonucleotides) or solid line (Seracare RNA Fusion v4 mix), and the copy number by color. RFU, relative fluorescence units.


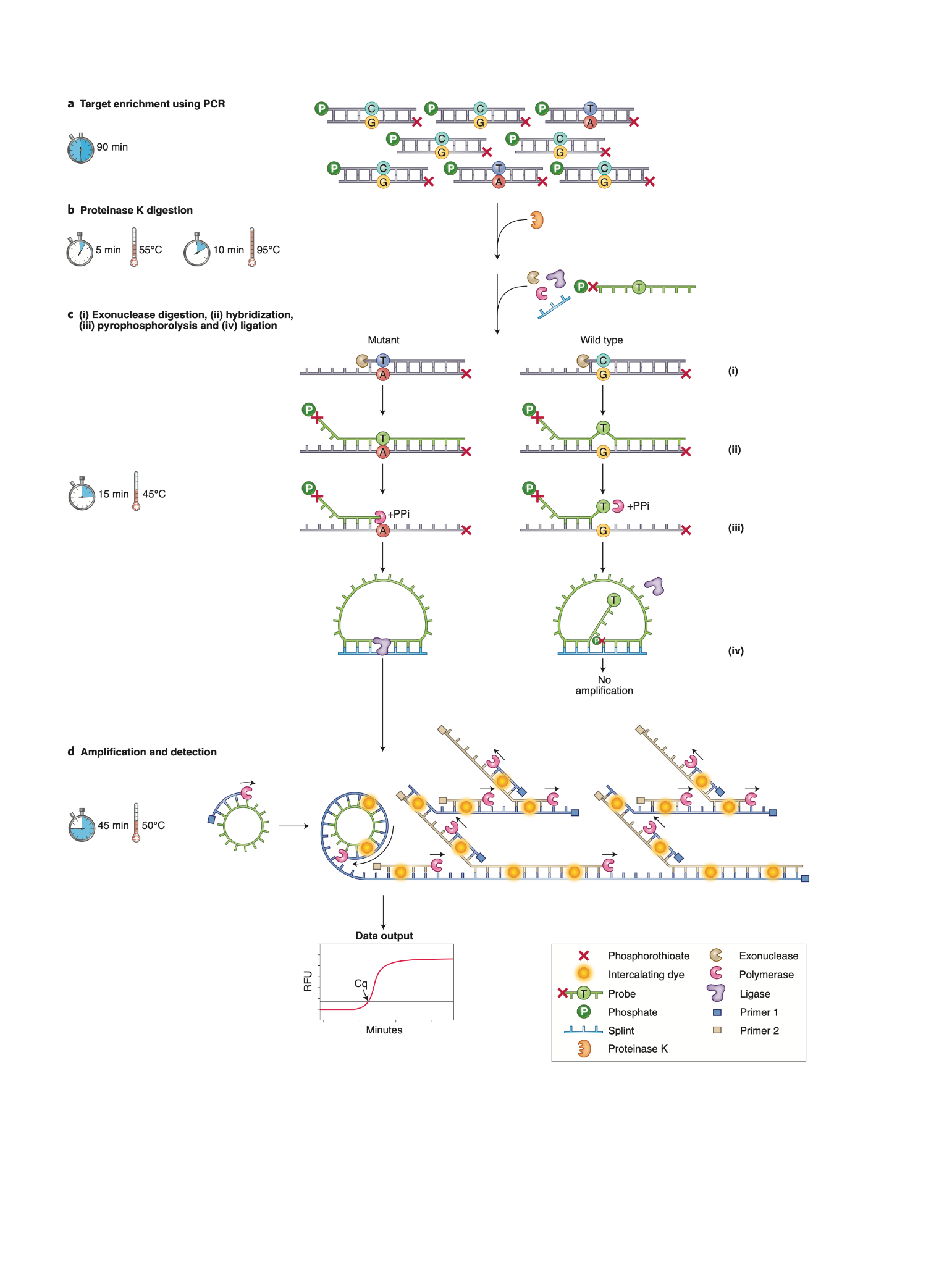


**Supplementary Figure 2:** Overview of the ASPYRE assay taken from [1]. (a) Multiplex PCR amplification of wild-type and mutant alleles. (b) Enzymatic digestion of the PCR enzyme and subsequent heat-inactivation. (c) Exonuclease digestion to create single-stranded target molecules; hybridization of oligonucleotide probes to both mutant and wild- type sequences, with a mismatch to the wild-type sequence at the site of mutation; and pyrophosphorolysis of hybridized probes. (d) Circularization of only those probes that have been digested beyond the mutation site; and isothermal amplification and detection of circularized probes. Artwork by Debbie Maizels, Zoobotanica Scientific Illustration.
